# An assessment of energy storage options for large-scale PV-RO desalination in the extended Mediterranean region

**DOI:** 10.1038/s41598-019-52582-y

**Published:** 2019-11-07

**Authors:** D. Ganora, C. Dorati, T. A. Huld, A. Udias, A. Pistocchi

**Affiliations:** 1 European Commission, DG Joint Research Centre, Dir. D – Sustainable Resources, Marine and Water Resources Unit. Via E.Fermi, 2749 – 21027 Ispra (VA), Italy; 2 Department of Environment, Land and Infrastructure Engineering, Politecnico di Torino, Corso Duca degli Abruzzi 24, 10129 Torino, Italy; 3 ARHS Italia, External Consultant for the European Commission, Joint Research Centre, Ispra, Italy

**Keywords:** Environmental sciences, Hydrology, Energy infrastructure

## Abstract

Seawater desalination is already a largely adopted option to cope with the scarcity of natural water resources, but the increasing concerns about water availability in the future make it even more attractive. Because desalination is a highly energy-demanding process, its coupling with renewable energy sources is an essential step for the sustainable production of desalinated water at large scales. In this work we analyze the potential to deploy large-scale seawater desalination using reverse osmosis (RO) under the hypothesis that all the required energy is provided by photovoltaic (PV) production. A simulation over the extended Mediterranean area shows that securing desalinated water for up to about 200 million people in the region is technically possible using PV only, and the benefits of energy storage in batteries and/or water reservoirs are usually higher than its costs. This suggests that water management policies could consider desalination more broadly and encourage PV-based RO, as a possible win-win and cost-effective strategy to improve water and energy resources security.

## Introduction

The growing need for freshwater resources, and increasing concerns about water availability in the future, are making seawater desalination an attractive option in many regions worldwide. Some countries have already installed a considerable desalination capacity to cope with systematic water shortages (e.g.^1^,), but many other countries are likely to follow as more and more affordable technological solutions become available.

Currently, the technology most commonly used for large-volume seawater desalination is reverse osmosis (RO)^1–3^ that overcame thermal technologies in the last decades. While more efficient than in the past, RO desalination is a highly energy-demanding process, with state-of-the-art technologies requiring between 2 and 4 kWh of electricity for the production of 1m^3^ of clean water^4,5^. If fossil fuels are used to produce the required energy, the carbon footprint of RO desalination is consequently high, making it non-sustainable.

Although it has been shown that RO desalination can be fully fed with renewable energy sources^6^, the intermittency of these sources requires to store energy in order to ensure continuous water production. In principle, electricity can be stored through the power grid; however, where the grid is not available (e.g., small islands) or has structural constraints (which may often be the case when a large power generation or demand is localized on a grid segment), it may be important to limit the exchanges with the grid through partial off-grid energy storage. This may even become critical when thinking desalination as a main source of freshwater over a region.

The aim of this work is to analyze the implications of large-scale RO desalination, if all the required energy is to come from renewable sources. We quantify the potential to deploy seawater RO desalination over the extended Mediterranean region considering photovoltaic (PV) as the most commonly accessible renewable energy source (e.g^3^.). In the analysis, we consider standard and well-established technologies, with typical parametrizations; detailed plant optimization is instead out of the scope of the paper. We consider the about 6,000 sites of potential desalination plants identified in^7^, and for each site we compute the PV capacity that we need to install in order to fully feed a RO desalination plant. The variability of available solar radiation at the site determines the energy storage needs in order to ensure a scheduled production of clean water, either with at-plant facilities (batteries and pumped-water reservoirs) or through exchanges with the grid.

We present and discuss the following performance indicators under different plant configurations: (1) percent of the time when the plant can be run without requiring power from the grid (plant autonomy); (2) the total energy exchanged with the grid (a proxy for the cost of grid use); (3) the statistics of power exchanges with the grid (a proxy of the impact of large-scale desalination on the grid); and (4) the size of the required at-plant energy storage.

Based on the results, we discuss the possibilities and challenges towards deploying seawater RO desalination in the Mediterranean using electricity coming 100% from PV.

## Methods

### PV-RO plant layouts considered in this study

The analysis is based on the simulation of schematic PV-powered RO plants using a deterministic algorithm described in further details in the supporting information (SI). The simulation is performed at hourly step considering a time series of PV producibility based on^8^. In our calculations, we refer to a single-stage RO unit, with a feed flowrate *Q*_*F*_ variable in time, to produce clean water with a flowrate *Q*_*P*_ = *RQ*_*F*_, where *R* is the recovery rate assumed to be 0.5 (see a typical layout and parameterization in [^4^, figure 3.17]). The feed pressure Π_*F*_ (bars) is computed in a simplified way^4,9^, as:

1 $${\Pi }_{F}=0.77\cdot \frac{{C}_{F}}{1-R}+{\Pi }_{{\rm{drop}}},$$

where *C*_*F*_ is the inflow water salinity (g/L) and Π_drop_ is the pressure loss within the RO unit, assumed equal to 4 bar^10^. The plant is assumed to operate with an energy recovery device (ERD) allowing the recovery of energy from high-pressure concentrate (e.g.^10,11^,).

In order to provide the feed pressure Π_*F*_, we consider two possible configurations: (1) a single high-pressure pump; (2) a water reservoir located at an elevation *H*_RES_, providing a pressure Π_RES _= *γ*_*S*_*H*_RES_ (where *γ*_*S*_ is the specific weight of seawater), followed by a booster pump providing an additional pressure Π_BOOST_ such that Π_*F *_= Π_RES _+ Π_BOOST_. In this case, the reservoir can receive as input a flow rate *Q*_*RES*_ variable in time, whose instantaneous value is different from *Q*_*F*_, although over a representative time of operation of the plant, *T*, it must satisfy:

2 $${\int }_{0}^{T}{Q}_{{\rm{RES}}}(t){\rm{d}}t={\int }_{0}^{T}{Q}_{{\rm{F}}}(t){\rm{d}}t.$$

Under configuration (1) the power required for the single high-pressure pump is

3 $${W}_{{\rm{P}}}=\frac{{Q}_{F}{\Pi }_{{\rm{F}}}(1-ER)}{{\eta }_{P}}.$$

Under configuration (2) the power required to pump seawater to the reservoir is approximated as

4 $${W}_{{\rm{RES}}}=\frac{{Q}_{RES}{\Pi }_{{\rm{RES}}}}{{\eta }_{P}},$$

while the power required for the boost pump is

5 $${W}_{{\rm{BOOST}}}=\frac{{Q}_{F}({\Pi }_{{\rm{BOOST}}}-{\Pi }_{F}\,ER)}{{\eta }_{P}},$$

where $ER=\frac{{\Pi }_{F}-{\Pi }_{{\rm{drop}}}}{{\Pi }_{F}}(1-R){\eta }_{ERD}$ is the recovery rate of energy from the concentrate, *η*_*ERD*_ being the ERD efficiency assumed to be 90%, and *η*_*P*_ the pumping efficiency assumed to be 85%. Finally, we considered an additional energy requirement for the pre/post-treatment of water, proportional to the pumping flow rates, through a proportionality constant α assumed to be 1 kWh/(m^3^d^−1^) according to the values reported by^5^. The power required to operate the RO plant is consequently given by:

6 $$\begin{array}{c}P(t)={{\rm{W}}}_{P}+\frac{\alpha \,R\,{Q}_{F}}{24}({\rm{for}}\,{\rm{a}}\,{\rm{single}}\,{\rm{pump}}\,{\rm{configuration}})\\ P(t)={{\rm{W}}}_{RES}+{{\rm{W}}}_{BOOST}+\frac{\frac{\alpha }{2}\,R\,({Q}_{F}+{Q}_{RES})}{24}\,({\rm{for}}\,{\rm{a}}\,{\rm{reservoir}}+{\rm{booster}}\,{\rm{pump}}\,{\rm{configuration}})\end{array}$$

Here we consider that the energy to run the process is entirely provided by a dedicated PV plant. The latter generates power following the diel and seasonal variation of solar radiation. The power that can be produced by a PV plant of nominal capacity *C*_*PV*_ (kW_p_) is estimated on the basis of the time series of PV producibility estimated by^8^. Power production reads *P*_*PV*_ = *C*_*PV*_*w*(*t*), where *w*(*t*) is the electric power that can be produced at time step *t* by a PV plant of 1 kW nominal power, with south-oriented panels with an optimal slope angle and 14% system losses. The capacity *C*_*PV*_ required to fully feed a given RO plant can be computed as:

7 $${C}_{PV}=\frac{{\int }_{0}^{T}P(t){\rm{d}}t}{{\int }_{0}^{T}w(t){\rm{d}}t}$$

In this study, we assume *T* to coincide with the length of the time series of PV producibility. Clean water production is scheduled following a modular pattern, where during each month *Q*_F_ is constant and proportional to the average value of *P*_*PV*_ for that month calculated over the whole time series of *w*(*t*), and we impose a yearly average of *Q*_F_ = 2 m^3^d^−1^ (i.e., *Q*_P _= 1 m^3^d^−1^) in order to obtain results referred to a unit production capacity. Under the assumed modular scheduling, we checked that maximum water production is typically about 2 times the minimum production rate (with values ranging from about 1.25 to 4). This suggests the adopted design to be a good compromise between a plant with fully variable RO operation on direct gear with PV power generation, and a plant with constant production over time (which would require a much larger energy storage than the modular scheduling).

**Figure 1:**
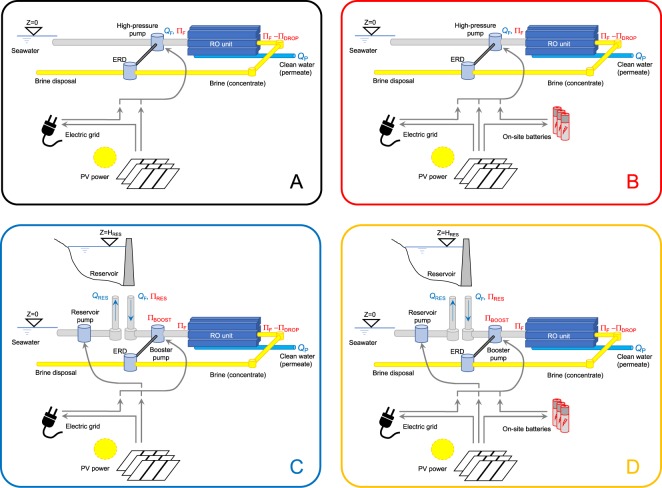
Layouts of PV-RO desalination plant used for simulation: (**A**) only grid to store energy; (**B**) grid + battery; (**C**) grid + reservoir; (**D**) grid + battery + reservoir. Q are flow rates and Π water pressure values at different points of the plant. ERD indicates the energy recovery device.

For what concerns energy storage, we consider two possibilities: (1) energy is only exchanged with the power grid, which provides electricity when PV production is not sufficient and receives the electricity produced by the PV plant when this exceeds the RO plant requirements; (2) part of the energy produced by the PV plant in excess of RO plant requirements is stored in a battery at the plant. Depending on the combination of hydraulic (single pump or reservoir + booster pump) and energy storage configurations, we have 4 possible layouts (“A”, “B”, “C”, “D”) of a combined PV-RO plant, as schematically illustrated in Fig. 1.

Under layouts “C” and “D” (reservoir + booster pump with grid exchange only or with battery) the reservoir is assumed to never be completely empty, so that it can always provide the required flow rate at pressure Π_RES_ (see SI, for more details). Moreover, the energy required to pump flow rate *Q*_*RES*_ into the reservoir is assumed to be provided only by direct PV power, so that grid and battery are never used to increase water storage. When enough PV power is available, it is first used for the single pump (under layouts “A” and “B”) or the booster pump (under layouts “C” and “D”). PV power in excess of the requirements of these pumps is allocated according to these hierarchical rules:

- under layout “A”, power in excess of the single pump is integrally fed to the grid;
- under layout “B” the battery is first charged with a limit on recharge power (corresponding to power used by the single pump or booster pump during maximum production), and any residual power is integrally fed to the grid;
- under layouts “C”, power is used first to pump water to the reservoir up to the maximum volume required to guarantee the *Q*_F_ flow and then fed to the grid;
- under layout “D”, the battery is first charged as in “B”; residual power, if any, is used first to pump water to the reservoir up to the maximum volume required to guarantee the *Q*_F_ flow and then fed to the grid.

During periods with scarce or no PV production, the single pump or booster pump is operated using electricity stored in the battery as long as available, and then electricity from the grid, while pressure Π_RES_ is guaranteed by gravity. For the sake of simplicity, energy losses in the battery and grid exchange are neglected.

### Study region

**Figure 2:**
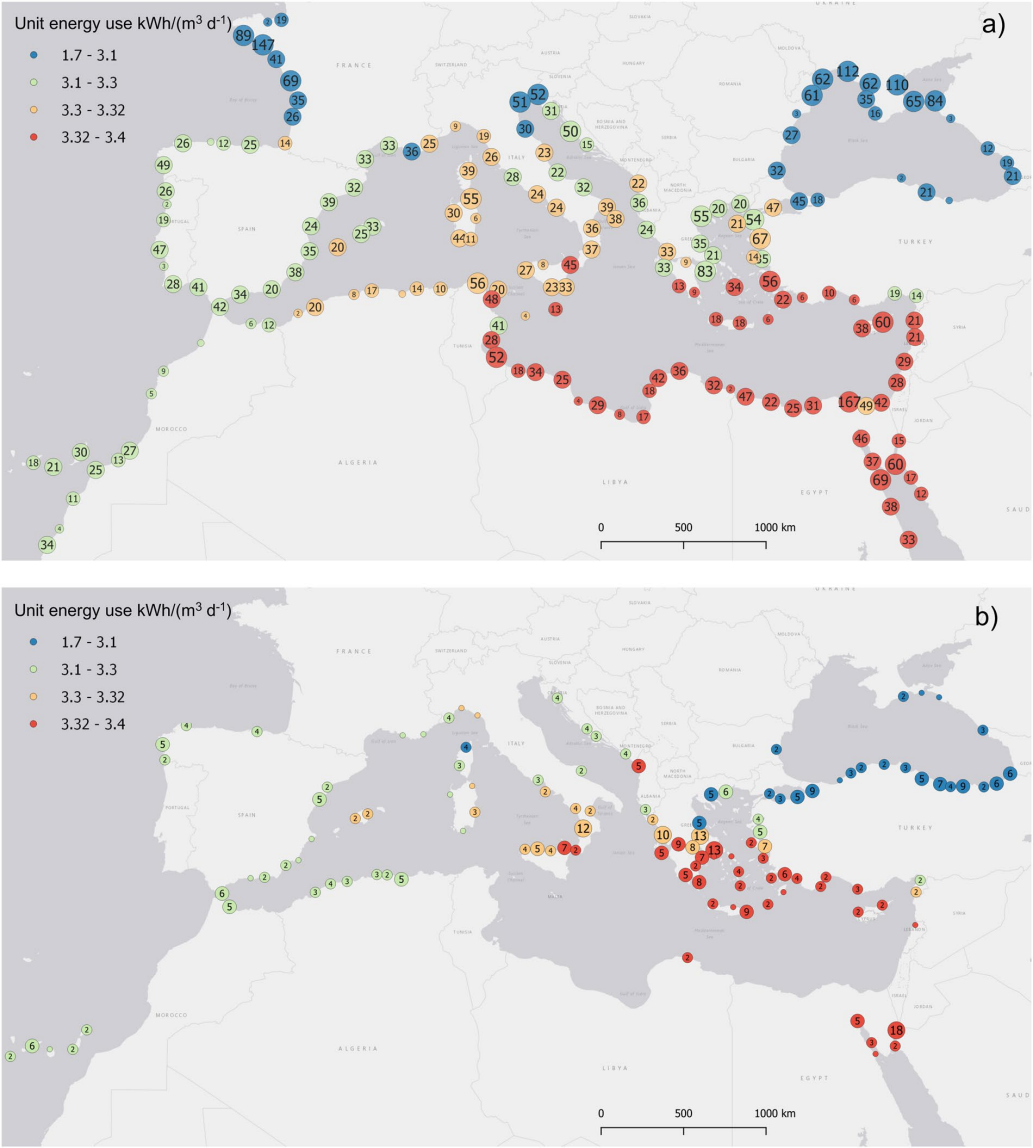
Map of the 5,927 potential plants (**a**), clustered by geographical proximity (the number indicates how many plant sites are located around the circle). The color scale shows the required unit energy use that includes pre/post treatment energy. (**b**) Plant sites where desalination can be potentially driven by the sole pressure of the reservoir according to the elevation values obtained with a 2 km search radius.

The above schematizations of PV-powered RO plants have been used to investigate the applicability of large-scale PV-powered desalination over a geographical area that includes the Mediterranean Sea, the Black Sea, the upper Red Sea, and the Atlantic coastline of Northern Africa and Southern Europe as in Fig. 2. The area has between 100 and 200 million people that could be serviced with desalinated water at affordable costs of transport^7^. Potential sites for RO desalination plants are selected for their favorable topography and land cover^7^, resulting in a set of 5,927 potential locations. Most of the sites (70%) have a seawater salinity larger than 36 g/l. Intermediate salinity (18–36 g/l) is present in 17% of the site, while sites on the Black sea have salinity equal or lower than 18 g/l. For each site we consider the time series of PV producibility in the period January 2006-December 2015 from^12^ based on^8^. Reservoir elevation *H*_RES_ is estimated as the maximum elevation found within a search radius of 1 km around the plant location; the analysis is also repeated considering the maximum elevation within 2 km radius.

For each site, we compute the following quantities:

- energy required for desalination (depending on the salinity of seawater) and relative contribution of the reservoir’s potential energy;
- capacity of the PV plant, *C*_*PV*_;
- under layouts “B” and “D”, the size of the battery (kWh/(m^3^ d^−1^)) required on the basis of the battery charge level experienced by the system during the simulated period;
- under layouts “C” and “D”, the size of the reservoir (m^3^/(m^3^ d^−1^)) required on the basis of the reservoir storage volume experienced by the system during the simulated period;
- under layouts “B”, “C” and “D”, the percent of time that the RO plant can run without exchanges with the grid compared to the bottom line of layout “A” (without energy storage);
- under all layouts, total energy and power exchanged with the grid (fed to and withdrawn from).

## Results

### Energy requirements and relative contribution of reservoir potential energy

The (cumulative) frequency distribution of required unit energy is provided in Table 1, while Fig. 2a shows its geographical variability within the case study. The median energy requirement is about 3.3 kWh/m^3^, while sites with the lowest requirements feature less than 2 kWh/m^3^. However, 75% of the sites has requirements in the narrow range of 3.1 to 3.4 kWh/m^3^. Table 1 reports also the relative contribution of the reservoir in terms of power: while the topography (and thus the reservoir elevation) is quite variable in the investigated area, in 41% of the sites the reservoir can provide more that 30% of the required energy and in 550 cases (Fig. 2b) the plants are, in principle, completely self-sufficient (i.e., RO completely driven by reservoir pressure) for 2 km of search radius (self-sufficiency reduces to 45 cases with 1 km of search radius). For example, a feed pressure of 57.9 bar is obtained (Eq. 1) for salinity 35 g/L (*R* = 0.5, Π_*drop*_ =4 bar); then the equivalent pressure to be provided net of the contribution of energy recovery is Π_*F*_(1−*ER*) that, with *ER* = 0.42, leads to 33.6 bar, i.e, about 291 m of seawater head. A topographic elevation of 100 to 150 m is not negligible compared to this total head.

**Table 1:** Overall frequency distribution (percentiles) of key plant parameters for search radius 1 and 2 km and considering pre/post-treatment energy for the 5,927 potential locations.

frequency distribution	Unit energy (RO + pre/post-treatment)	*W*_RES_/(*W*_RES +_ *W*_BOOST_)		PV Capacity
percentile	kWh/(m^3^d^−1^)	1 km	2 km	kW_P_
1%	1.89	0.009	0.012	0.51
5%	1.99	0.023	0.032	0.55
10%	2.16	0.034	0.044	0.57
25%	3.13	0.063	0.090	0.63
50%	3.29	0.142	0.219	0.71
75%	3.32	0.301	0.507	0.76
90%	3.35	0.488	0.883	0.81
95%	3.37	0.624	self suff.	0.85
99%	3.37	0.911	self suff.	0.97

### PV capacity requirements and on-site energy storage

The production of 1 m^3^ of clean water per day requires to install a nominal capacity that depend on the PV productivity as described in Eq. (7). A median value of 0.71 kW_p_ has been obtained over the case study, with most of the potential plants resulting in values lower that 0.83 kW_p_. The overall frequency distribution of the capacity is reported in Table 1.

The design size of the battery and reservoir have been computed as the 95^th^ percentile of the empirical frequency curve of the hourly battery charge level and the hourly reservoir water storage, respectively. This percentile-based size can be considered more robust with respect to the absolute maximum values, which are highly influenced by sporadic critical conditions. Battery and reservoir design size values are summarized in Table 2 for layouts “B”, “C” and “D” (reporting the percentiles of their frequency distribution). We must highlight that, due to the power allocation rules, the joint use of battery and reservoir (layout “D”) causes a reduction of battery capacities, and an increase of reservoir volumes compared to the case of batteries or reservoirs alone.

**Table 2:** Overall frequency distribution (percentiles) of the design battery and reservoir size, and of the peak power fluxes to the grid for the different setups (A = grid only; B = grid + battery; C = grid + reservoir; D = grid + battery + reservoir).

layout	“B”	“C”	“D”		“A”	“B”	“C”	“D”	“A”	“B”	“C”	“D”
percentile	Batt. size kWh/(m^3^d^−1^)	Res. size m^3^/(m^3^d^−1^)	Batt. size kWh/(m^3^d^−1^)	Res. size m^3^/(m^3^d^−1^)	Peak power flux from grid kW/(m^3^d^−1^)				Peak power flux to grid kW/(m^3^d^−1^)			
1%	2.16	1.21	0.32	1.26	2.79	2.54	1.57	1.68	6.81	3.99	2.69	0.41
5%	2.25	1.25	0.63	1.36	2.99	2.76	1.91	1.96	7.29	4.26	4.35	1.63
10%	2.28	1.32	0.74	1.58	3.14	2.88	2.10	2.13	7.69	4.44	4.97	2.13
25%	2.34	1.77	0.86	3.19	3.67	3.27	2.45	2.52	8.47	4.77	5.86	2.75
50%	2.39	2.98	0.98	5.52	4.04	3.73	2.96	2.97	9.48	5.38	6.88	3.43
75%	2.45	4.27	1.06	9.13	4.33	3.95	3.17	3.17	10.22	5.88	7.71	3.97
90%	2.49	5.58	1.14	14.11	4.48	4.15	3.38	3.39	10.98	6.70	8.62	4.68
95%	2.53	6.75	1.17	17.54	4.57	4.29	3.50	3.51	11.52	7.34	9.27	5.23
99%	2.58	14.56	1.25	37.12	4.70	4.43	3.65	3.68	12.68	8.62	9.71	5.65

### Exchanges with the grid

**Figure 3:**
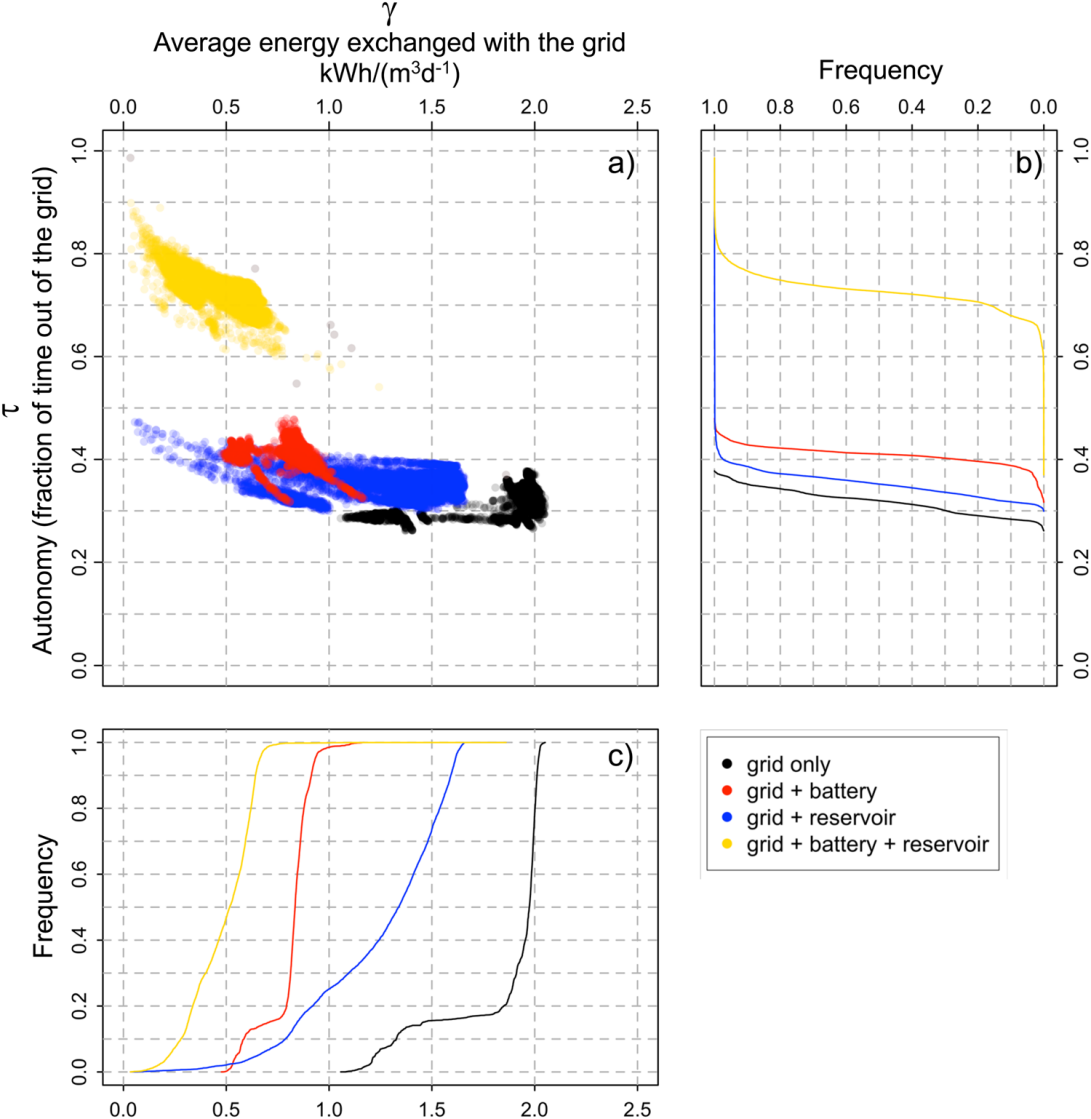
Comparison of plant autonomy *τ* and energy exchange with the grid *γ* for the whole dataset (**a**), for the different plant layouts and considering 1 km of search radius and the additional pre/post-treatment energy use. Panel **b** reports the frequency distribution of *τ* and panel **c** the frequency distribution of *γ*.

Figure 3a shows the role of local energy storage options in terms of the autonomy of the plant (*τ*) (i.e., the percentage of time the plant is not adsorbing power from the grid) plotted against the average unit energy exchanged with the grid (*γ*) under the four layouts examined here. The marginal distributions of the two variables are represented as cumulates in panes b and c. The use of battery alone (layout “B”) allows a reduction of the median *γ* to 42% of the standard configuration (without energy storage) while the use of reservoir alone (layout “C”) to 68%. The joint use of reservoir and storage (layout “D”) improves the power exchange reducing its median value to 26% of that under layout “A”. On the other hand, layout “D” greatly enhances the autonomy of the plants from the grid with median *τ* around 0.7 while the best performing alternative option (layout “C”) hardly exceeds *τ* = 0.4. Similar considerations can be made for 2 km search radius although the performances of the reservoir are slightly improved (results not shown here for conciseness).

For each simulation we also recorded the time series of power to and from the grid, which may exceed the grid’s buffering capacity. It is worth noting that the instantaneous power exchanged with the grid is not symmetric: when the grid supplies power to the plant, the limit is the maximum power used by the booster pump and depends on the water production pattern and on seawater salinity. On the other hand, the PV production exceedance is supplied to the grid without any limitation; for the peak power sent to the grid, we consider the 95^th^ percentile of the empirical frequency curve of the hourly power in order to exclude extreme peaks. Peaks of power taken from the grid are for each setup bounded within approximately 1.5 and 4.5 kW/(m^3^d^−1^). Peaks supplied to the grid are instead more variable and can exceed in some cases the 10 kW/(m^3^d^−1^). Their overall frequency distribution is reported in Table 2 for the different setups: the combined use of battery and reservoir reduce the peaks to the grid to ranges similar to those from the grid. These peak values refer to power exchanges at the individual plant level, while we may expect the average of exchanges over several sites to have smaller fluctuations. In order to appraise this, we have evaluated a virtual scenario in which plants at all potential sites operate at the same production rate of clean water. Further, we make the hypothesis that all the plants are connected with an electric grid able to redistribute instantaneously the power among the plants. Under these ideal conditions, the net power peaks to the grid would reduce to 0.30 kW/(m^3^d^−1^) for layout “A”, 0.16 kW/(m^3^d^−1^) for “B”, 0.21 kW/(m^3^d^−1^) for “C” and 0.09 kW/(m^3^d^−1^) for “D”. These values are 1 to 2 order of magnitude lower than the typical net exchanges with the grid of individual plants (Table 2).

## Discussion and Conclusions

### Implications of the PV capacity required for the mediterranean region

According to the simulations, PV capacity has a median value of 0.71 kW per installed kW to produce 1 m^3^d^−1^ of clean water. If we extrapolate to the large-scale, considering a daily consumption of 0.2 m^3^ per person, the total PV capacity to install corresponding to 100–200 million people would be 14.2–28.4 GW. With the same extrapolation, assuming 7 m^2^ of solar panels required for a capacity of 1 kW the overall area needed to supply water for 100–200 million people would be approximately 100–200 km^2^, respectively.

### Costs of on-site energy storage to manage intermittency

**Figure 4:**
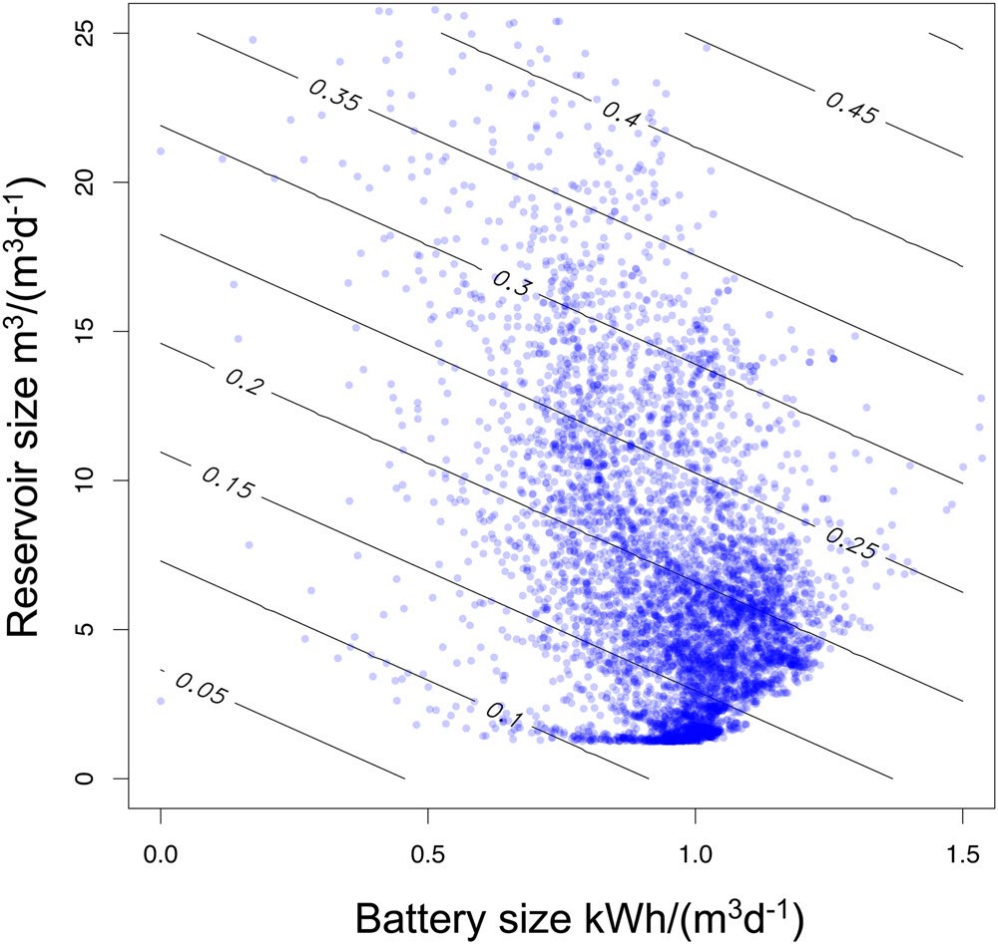
Battery vs reservoir preliminary design size and corresponding iso-lines of the cost for energy storage facilities. Unit costs considered are: 200 € per kWh/(m^3^d^−1^) with payback of 5 years for the battery and 50 € per m^3^/(m^3^d^−1^) with payback of 10 years for the reservoir. Reservoir search radius of 1 km.

Energy storage requires investments in batteries and/or reservoirs. A preliminary evaluation of this can be made assuming a unit cost of the battery of 200 €/kWh and a payback period of 5 years and a unit cost of the reservoir of 50 €/m^3^ and a payback period of 10 years (conservative values compared to those used in^13^). Although these values do not reflect the actual variability of the local costs over the study area (particularly important for reservoirs) and do not account for economies of scale, they allow a cost-based ranking of the plants, highlighting sites where the energy storage is potentially feasible. The levelized cost of these investments (Euro/m^3^ of clean water produced) can therefore be estimated as

$$\frac{200\,\times {C}_{B}}{365\,\times \,5}+\frac{50\,\times {C}_{R}}{365\,\times 10},$$

where *C*_*B*_ and *C*_*R*_ are the capacities of batteries (kWh/(m^3^ d^−1^)) and reservoirs (m^3^/(m^3^ d^−1^)). Figure 4 reports a plot of the storage and battery requirements under layout “D” for the about 6,000 sites analyzed, with the corresponding iso-cost lines superimposed. This application shows that, for 50% of the sites, energy storage may imply a cost equal to, or lower than 0.19 €/m^3^ of clean water produced. This cost is to be added to water production costs. A threshold cost of 0.23 €/m^3^ is not exceeded in 75% of the sites.

**Figure 5:**
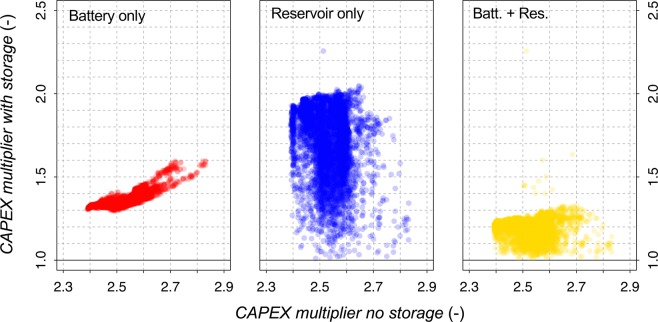
CAPEX multiplier to be considered for oversized plants in order that keep the same water production if the grid is absent or not reliable. The abscissa refers to the plant without energy storage; the ordinate refers to the three energy storage options including battery, reservoir or both.

Our estimate of about 0.2 €/m^3^ is the cost of energy storage in order to ensure continuity of water production, under the assumption that power can be anyway exchanged with the grid. It is interesting to evaluate the volume that can be produced by a given plant, when it is not possible to exchange power with the grid. The ratio *ϕ* between the volume produced without resorting to the grid and the volume produced with continuous operation of the modular scheduling is usually higher than the autonomy in terms of time, *τ*, due to the fact that, when some energy is required from the grid, there may be still some energy available from the PV system. Because the capital expenditure (CAPEX) for producing 1 m^3^ of water is obtained from the present value of the investment cost divided by the total production volume, if a plant does not produce to full capacity because it cannot rely on power from the grid, its CAPEX increases. Therefore, 1/*ϕ* represents the plant’s CAPEX multiplier due to lack of exchanges with the grid. Figure 5 compares the CAPEX multiplier (1/*ϕ*) in case of no storage (layout “A”) compared to the same value obtained from layouts “B”, “C” and “D”. Values around 2.5 in case of layout “A” mean that, without exchanging with the grid, the CAPEX of produced water would be 2.5 times higher than with grid exchanges. The CAPEX multiplier reduces to around 1.4 when using battery storage, to less than 2 (but with high variability) when using reservoir storage and to around 1.2 for the combined use of battery and reservoir.

This allows appreciating the possible benefits of energy storage for a plant. For instance, if the CAPEX under continuous operation (i.e., with grid exchanges) is 0.3 €/m^3^, the CAPEX of a plant without energy storage and no grid would be about 2.5 times higher, or 0.75 €/m^3^ in the absence of storage. The same plant with battery and reservoir storage would have a CAPEX 1.2 times higher, or 0.36 €/m^3^ plus the cost of storage typically around 0.2 €/m^3^, with a net cost reduction of 0.75–0.36–0.2 = 0.19 €/m^3^.

### Concluding remarks and perspectives

Securing water availability to millions of people is technically possible thanks to large-scale desalination but requires considering, besides direct environmental impacts such as brine disposal, the indirect impacts due to its large energy requirements. Coupling desalination with renewable energy sources is therefore essential for the sustainable production of desalinated water.

Based on the analysis presented here, we have shown that supplying desalinated water to 100–200 million people in the extended Mediterranean region requires a PV installed capacity of 14.2–28.4 GW, which is large but acceptable in the context. Where the power grid is adequate to completely buffer the intermittency of PV generation, the production of clean water may be scheduled at a constant rate. However, where the grid is not adequate, clean water production needs to adapt to the variability of power. A modular scheduling of production proportional to monthly average solar radiation, and the conjunctive use of on-site electric batteries and pumped reservoirs significantly enhance the autonomy of the plant (up to about 70% of the time of operation), although complete autonomy from the grid would require very large energy storage facilities, currently beyond economic feasibility. On-site energy storage also regulates power exchanges with the grid, thus reducing risks of overload of the electric system. The investments for the implementation of on-site energy storage are expected to add an order of 0.2 €/m^3^ to the cost of water production; from these costs, one should deduct the avoided costs of power exchange with the grid, strongly dependent on the local infrastructure and market conditions. If the grid exchange is not possible or unreliable, the plant would be operated intermittently leading to increased capital costs to achieve the same water production. Under this condition a plant without storage facilities can have overall capital costs larger than a similar plant with internal energy storage, although energy storage requires further investments.

Increasing the PV installed capacity beyond desalination requirements may be an alternative solution to improve the plant’s autonomy. Preliminary simulations (not shown here for conciseness) suggest that moderate PV oversizing (5–10% in excess of desalination requirements) may significantly improve plant autonomy because the excess generated power could provide extra recharge to the batteries. A systematic investigation of this aspect is anyway beyond the scope of this contribution. The solar energy in excess of desalination requirements may also offset the carbon footprint of other energy uses.

The average of the power exchanges with the grid of all plants in the region is obviously smaller than power exchanges at a single plant due to the cancelling out of individual peaks. This suggests that designing desalination plants as a regional network, rather than in isolation, may significantly stabilize the electric grid, and hints to the relevance of international cooperation on investments and the pooling of water and energy resources, as a possible win-win and cost-effective strategy to improve their security.

## Supplementary information

Simulation of PV-driven desalination with energy storage

## Data Availability

The analysis can be reproduced and applied elsewhere using the algorithm described in the SI.
