# Supplementary material for: An assessment of energy storage options for large-scale PV-RO desalination in the extended Mediterranean region
Source: Sci Rep. 2019 Nov 7;9:16234. doi: 10.1038/s41598-019-52582-y (PMC6838325; doi:10.1038/s41598-019-52582-y)
Supplement: Supplementary file 1 — Simulation of PV-driven desalination with energy storage [file 41598_2019_52582_MOESM1_ESM.docx]

# Simulation of PV-driven desalination with energy storage

## Supplementary material of the manuscript “An assessment of energy storage options for large-scale PV-RO desalination in the extended Mediterranean region”

D.Ganora^1,2*^, C.Dorati^3^, T.A.Huld^1^, A. Udias^1^, A.Pistocchi^1^

^1^ European Commission, DG Joint Research Centre, Dir. D – Sustainable Resources, Marine and Water Resources Unit. Via E.Fermi, 2749 – 21027, Ispra (VA), Italy.

^2^ Department of Environment, Land and Infrastructure Engineering, Politecnico di Torino, Corso Duca degli Abruzzi 24, 10129 Torino, Italy.

^3^ ARHS Italia, External Consultant for the European Commission, Joint Research Centre, Ispra, Italy.

[* daniele.ganora@polito.it](mailto:*%20daniele.ganora@polito.it)

## Abstract

The intermittency of renewable energies requires to store the produced energy to run industrial processes at a constant pace. This algorithm quantifies the opportunities and limits of energy storage for seawater desalination driven by photovoltaic production, with a specific focus on large-scale applications. It allows the estimation of energy fluxes occurring with different storage options: on-site electricity storage in batteries; on-site energy storage as pressure in a water reservoir; electricity storage through exchange with the power grid. Furthermore, the algorithm:

- allows the preliminary sizing of the storage capacities;
- support the testing of grid and batteries reliability providing the frequency distribution of energy exchange;
- allows the use of different pattern of desalinated water production.

## Keywords

Photovoltaic-assisted desalination; batteries; water storage; grid exchange; plant operation simulation

# Introduction

This paper describes the algorithm used by Ganora et al. (2019) to study different options for energy storage in desalination plants. The desalination plant is represented as a single reverse osmosis (RO) unit fed with a high-pressure seawater flow that produces a permeate flow of clean water and brine as byproduct of the process. The schematic layout of the plants is sketched in Figure 1a, while more details on technical aspects of desalination can be found in Voutchkov (2012). A different layout is obtained allowing (partial) energy storage by means of a reservoir (Figure 1b) where energy is stored as water pressure that directly contribute to the feed flow pressure in the RO unit. If the pressure provided by the reservoir is not enough to operate the RO unit, the feed flow pressure is increased to the correct value by a booster pump.

The system is considered fully fed by photovoltaic (PV) energy produced by a dedicated plant; the PV production that temporarily exceeds the plant consumption is supplied to the electric grid and returned to the plant when needed to operate the pumps. The exchange with the grid can be reduced by employing on-site batteries that buffer a share of the PV excess.

Overall, four different possible options for energy storage are possible: according to Ganora et al. (2019) we consider the layout “A” with no on-site storage, but only energy exchange with the grid; layout “B” with battery for on-site electricity storage, other than the grid; layout “C” with reservoir as energy storage, other than the grid; and layout “D” with battery, reservoir and grid. Note that the reservoir provides energy directly in form of pressure thanks to the hydraulic load, meaning that the contribution of the reservoir is intrinsically limited by the actual reservoir elevation that depends on the local topography.

# System definition

The model requires as input the time series of the feed flow rate, $Q_{F}$, and the time series of PV production $P_{PV}$, both discretized with time step $\Delta t$. Then, it simulates the time series of: (i) the power used by the reservoir pump and the reservoir storage level; (ii) the power fluxes to/from battery and the battery storage level; (iii) the power fluxes to/from the grid, from which various indicators are computed. Simulation results are used for preliminary sizing of energy storage elements and impact analysis.

The algorithm is general and can be applied to other contexts. However, it is worth noting that it has been primary intended to process a large number of possible plants and layouts, running simulations for long periods at fine time resolution, thus implying that the algorithm must be relatively fast and provide robust and reliable results. Hence, it may be not suitable for single-plant optimization. The procedure includes deterministic rules and is designed to close the overall energy balance on the long terms, i.e., at the end of the simulation all the produced energy is used for the desalination process. We assume that the feed flow rate $Q_{F}$ is known a priori (Ganora et al., 2019) according to water production needs. This allows one to quantify the overall required energy, the instantaneous power required by the high-pressure/booster pump and the amount of energy that must be stored. The actual energy storage level, instead, depends on the layout of the plant and the rules adopted to manage it.

Considering the layout of Figure 1a, the high-pressure pump used to feed the RO unit with a flow rate $Q_{F}$ has a power consumption $W_{HP}$. In the case of Figure 1b, the instantaneous power consumption is due to the simultaneous use of the booster and the reservoir pumps, the latter working with a flow rate $Q_{RES}$ that is in general different from $Q_{F}$. The actual power used by the pumps is thus $W_{RES}+W_{BOOST}$ (see Ganora et al., 2019, for details). To evaluate the energy storage capabilities of the reservoir, it is convenient to define the pressure-equivalent power provided by the reservoir, $W_{RES,F}$, that is equal to the power of the reservoir pump operated at the flow rate $Q_{F}$.

Additional energy for pre- and post-treatment of water is usually considered; to model this case we defined an equivalent power used by the pump, which reads $P_{HP}=W_{HP}+\alpha R\frac{Q_{F}}{24}$ in the case of layout “A” and “B”, and $P_{BOOST}=W_{BOOST}+\frac{\alpha}{2}R\frac{Q_{F}}{24}$ and $P_{RES}=W_{RES}+\frac{\alpha}{2}R\frac{Q_{RES}}{24}$ in the case of layout “C” and “D”, being $\alpha$ the unit pre/post-treatment energy (assumed equal to 1 kWh/(m^3^d^-1^) in this work).

# Algorithm implementation

The algorithm is initialized with empty battery and reservoir and run sequentially. Power production from the PV plant is allocated according to the hierarchy of Figure 2 for the different layouts: for instance, referring to Figure 2d, the available power is primarily used for the booster pump, then to charge the battery and only the excess is used to fill the reservoir and exchange with the grid; moreover, batteries cannot be used to pump water to the reservoir. The following sections describe how the algorithm works for the most complex layout “D”, but the other options can be obtained by excluding the desired energy storage element.

## Power allocation for the booster pump

The booster pump is considered always in operation at the flow rate $Q_{F}$ with electricity consumption $P_{BOOST}$ that is provided by either the PV production or the energy storage (grid or battery). The power flux directly used from PV to run the booster pump is

$a\left( i \right)= min\left[ P_{BOOST}\left( i \right), P_{PV}\left( i \right) \right]\text{ }\forall\text{ }i$,

that is limited by either the pump request or the PV production. To keep the booster pump continuously in operation, when $P_{PV}<P_{BOOST}$ the sole PV production is not sufficient and must be complemented with the stored electric energy, providing the power

$$b\left( i \right)= P_{BOOST}\left( i \right)-a\left( i \right)\text{ }\forall\text{ }i.$$

Note that $b$ is bounded with maximum value equal to the maximum of $P_{BOOST}$ when PV production is null (e.g., during night time).

We then consider the time series $c$, defined as the direct PV power used to pump the feed flow rate $Q_{F}$ to the reservoir, which reads

$c\left( i \right)= min\left[ P_{PV}(i)-a(i), P_{RES,F}\left( i \right) \right]\text{ }\forall\text{ }i$,

where $P_{PV}(i)-a\left( i \right)$ is the residual power net of the direct use of the booster pump. Note that in general the reservoir pump operates with a flow rate greater than $Q_{F}$ when PV power is available; $c$ should be interpreted as the power used to keep the base flow rate without water storage. The overall power excess from direct use,

$e\left( i \right)=P_{PV}\left( i \right)-a\left( i \right)-c\left( t \right)$,

is the function to be buffered through energy storage to overcome power deficiency during period when $P_{PV}$ is not sufficient.

The first energy storage option is the on-site battery; we assume that: i) it can be used only to power the booster pump, ii) that its charging/discharging rate is limited to the maximum value that can be absorbed by the buster pump, and iii) that has a maximum capacity $E_{BATT}^{Max}$. Let $P_{BATT}$ be the power flux to the battery (positive values for recharge, negative values for discharge) and $E_{BATT}$the actual charge level of the battery we compute for each temporal step the time series of recharge/discharge as

$$\left\{ \begin{aligned} P_{BATT}\left( i \right)=\min\left[ \begin{matrix} e\left( i \right) \\ \max\left[ P_{BOOST} \right] \\ \frac{E_{BATT}^{Max}-E_{BATT}\left( i \right)}{\Delta t} \end{matrix} \right]-\min\left[ \begin{matrix} b\left( i \right) \\ \max\left[ P_{BOOST} \right] \\ \frac{E_{BATT}\left( i \right)-0}{\Delta t} \end{matrix} \right] \\ E_{BATT}\left( i+1 \right)=E_{BATT}\left( i \right)+P_{BATT}\left( i \right) \Delta t \end{aligned} \right.$$

where the above equation reports all the considered constraints. In particular, the first min operator quantifies the recharge flux that can be limited by either: the available power form PV; the maximum allowed flux to the battery; the maximum storage capacity of the battery. If the battery is supposed to have no upper storage limits, the latter constrain can be set to a very large value. On the other hand, the second min operator quantifies the battery discharge flux that can be limited by either: the required power from the booster pump; the maximum allowed flux from the battery; the available stored energy in the battery before depletion. Energy flux to/from battery and battery charge level can be determined as sequential operations. Finally, the power $b\left( i \right)-P_{BATT}(i)$ is required from the grid to power the booster pump. However, the power provided to the grid can be determined only after the definition of the pattern of energy storage in the reservoir.

## Power allocation to the reservoir pump

The reservoir pump allows to store, as water pressure, a part of the energy required for the RO; it cannot be operated using battery or grid storage except during the initialization of the simulation, when the reservoir is empty. In this case the reservoir pump is operated at flow rate $Q_{F}$ with power from the grid for the time needed to activate the water storage.

As defined above, $c$ is the power-equivalent reservoir outflow that does not increase the storage, being instantaneously replaced by water provided by the reservoir pump. Here we introduce the complementary variable $d$ that defines the power-equivalent outflow from the reservoir during periods of scarce or no PV production. In practice, $c+d=P_{RES,F}$ represents the reservoir contribution in terms of power; however, $c$ is directly provided during PV-rich periods, while $d$ must be stored as water volumes and released during PV-poor periods. This hypothesis implies that the reservoir never stays empty (while battery can do). $d$ is computed as

$d\left( i \right)= P_{RES,F}\left( i \right)-c\left( i \right)\text{ }\forall\text{ }i$,

where the time series of $d$ is completely defined by the input data. The residual power availability, after removing from the PV production the direct use and energy allocated to the battery, is defined as:

$f(i)=e(i)-\max\left[ P_{BATT}\left( i \right), 0 \right]\text{ }\forall\text{ }i$.

For each time step, $f$ is split into two components $P_{RES}$ and $P_{GRID}$: the former allows the buffering of the pressure energy by running the reservoir pump; the latter counterbalances the energy exchange with the grid used to support the booster pump when the battery is empty. In general, the sharing between $P_{RES}$ and $P_{GRID}$ requires further hypotheses to be determined.

To make the problem solvable in a deterministic way we assume that during plant operation is possible to perfectly predict the time series of PV; being also $d$ perfectly known, it is possible to allocate in advance the exact amount of energy-equivalent water to satisfy the equivalent power $d$. The procedure can be summarized as follows:

- The time series of $f$ is segmented into a set of storage-depletion periods during which the reservoir accumulates an amount of energy and releases it completely (Figure 3a). The segmentation process can be conveniently done considering the time-cumulative energy of the difference between $f$and $d$ (red line in Figure 3b); each *k*-th period is identified between each local minima $m$ (red dots in Figure 3b) starting from the last value and moving backward.
- For each *k*-th period the energy that must be stored in the reservoir is $M_{k}-m_{k}$, while the residual energy that will be stored in the grid is $m_{k}-m_{k-1}$.
- $f$ is split between reservoir pump and grid proportionally according to the rule

$$\begin{matrix} P_{RES}\left( i \right)=\rho_{k} f\left( i \right) \\ P_{GRID}\left( i \right)={(1-\rho}_{k}) f\left( i \right) \end{matrix}$$

with $\rho_{k}=\frac{M_{k}-m_{k}}{m_{k}-m_{k-1}}$.

- Figure 3c reports, as stacked values, the actual power allocated to the reservoir (blue line) and the power to the grid (green line).
- The water flow rate of the reservoir pump is computed from $P_{RES}$(and the pre-treatment consumption) by means of the pump equation

$$Q_{RES}=\frac{W_{RES}\eta_{P}}{\Pi_{RES}}$$

where $\eta_{P}$ is the pump efficiency and $\Pi_{RES}$ the pump pressure.

- The volume stored in the reservoir is computed integrating $Q_{RES}$over time.

# Application and conclusions

The algorithm has been applied to analyze the different energy storage options for 5,927 potential plants (Pistocchi et al., 2018). Hourly time series of PV production, $P_{PV}$, have been obtained from the PVGis portal (PVGis team, 2017; see also Huld and Amillo, 2015) for the period January 2006-December 2015. The input feed flow, $Q_{F}$, is defined to be 2 m^3^d^-1^ (i.e., 1 m^3^d^-1^ of desalinated water) on average, with a monthly variability proportional to (long-term) monthly average solar radiation. An upper limit of battery storage of 80 kWh has been considered to control a minority of cases that would result in extremely large and unsuitable batteries.

Scope, hypotheses and overall results of the analysis are detailed in Ganora et al. (2019), while here an example relative to a single potential plant is reported for the layout “D”. A first result of the analysis is the time series of the most important variables; an example is reported in Figure 4, where a subset of the records (five days at hourly time scale) is reported. Panel a reports the produced PV power, while the actual power used by the pumps can be computed as sum of values in panel b (booster pump) and c (reservoir pump). The buster pump uses a constant power because in the reported period the feed flow is constant, while the reservoir pump operates when power is available. Panels e-f show the power fluxes to the energy storage facilities (positive values) and the power retrieved from the storage (negative values).

Overall results can be summarized by considering the empirical frequency distribution of the whole records as reported in Figure 5. Panel a highlights that the booster pump is always in operation; the step-behavior of the curve is due to the monthly-based feed flow variability. On the other hand, the reservoir pump is turned off for 65% of the time because is operated only by direct use of PV power. Figure 5b shows the power fluxes to/from battery and grid: it is worth noting that the power to the grid is not limited and larger values may be relevant if the grid has limited capacity. Finally, panels c and d show the distribution of the energy storage levels experienced by the battery and reservoir, respectively. These curves have been used to define the preliminary design size of the battery and reservoir as their 95^th^ percentile value. This percentile-based size, being less influenced by sporadic extreme values, can be considered a robust first-try estimator of the design size.

# References

Ganora, D., Dorati, C., Huld, T.A., Udias, A., Pistocchi, A., (2019) An assessment of energy storage options for large-scale PV-RO desalination in the extended Mediterranean region, submitted to *Scientific Reports*

Huld, T., Gracia-Amillo, A., (2015) Estimating PV Module Performance over Large Geographical Regions: The Role of Irradiance, Air Temperature, Wind Speed and Solar Spectrum, *Energies*, 8, 5159-5181

Pistocchi, A., Dorati, C., Huld, T.A., Salas Herrero, F., (2018) *Hydro-economic assessment of the potential of PV-RO desalinated seawater supply in the Mediterranean region: Modelling concept and analysis of water transport costs*, EUR 28982 EN, Publications Office of the European Union, Luxembourg, ISBN 978-92-79-77211-5, doi:10.2760/8455, JRC109866

PVGis team, (2017) Photovoltaic Geographical Information System (PVGIS) - Geographical Assessment of Solar Resource and Performance of Photovoltaic Technology. <http://re.jrc.ec.europa.eu/pvgis.html> (last access: April 29, 2019)

Voutchkov, N. (2012). *Desalination engineering: planning and design*. McGraw Hill Professional. ISBN: 978-0-07-177716-2

Figure 1 Layout of the plant with only the high-pressure pump (a) and with the reservoir as energy storage (b).

Figure 2 Hierarchy of PV electricity use (from top to bottom) with different energy storage options: a) grid only; b) battery + grid; c) reservoir + grid; d) battery + reservoir + grid.

Figure 3 Rule for power allocation to the reservoir and the grid: a) the residual power to be split between reservoir and grid and the equivalent power that is expected to be provided by the reservoir; b) segmentation of the record in period with complete storage-depletion of the reservoir; c) final time series of allocated power. All the variables refer to an average production of 1m^3^/d of desalinated water

Figure 4 An example of time series of the power production (a), power used by the pumps (b and c) and power exchange with the battery (d), reservoir (e) and grid (f) for a plant with layout “D”. For the sake of clarity, only a 5-day subset of the record is reported. All the variables refer to an average production of 1m^3^/d of desalinated water

Figure 5 Frequency distribution of the hourly values of the main variables over the whole period of analysis: a) power from PV and power used by the pumps; b) power flux to the battery/grid (positive values) and from the battery/grid (negative values); c) battery storage level; d) reservoir storage level. All the variables refer to an average production of 1m^3^/d of desalinated water.
